# Systematic reviews in Speech-Language Pathology

**DOI:** 10.1590/2317-1782/20212021170

**Published:** 2022-01-12

**Authors:** Vanessa Veis Ribeiro, Carla Patrícia Hernandez Alves Ribeiro César, Raphaela Barroso Guedes-Granzotti, Priscila de Oliveira, Kelly da Silva

**Affiliations:** 1 Departamento de Fonoaudiologia, Universidade Federal de Sergipe – UFS - Lagarto (SE), Brasil.; 2 Departamento de Fonoaudiologia, Universidade Federal de Sergipe – UFS -Aracaju (SE), Brasil.; 3 Departamento de Fonoaudiologia, Universidade Federal da Paraíba – UFPB - João Pessoa (PB), Brasil.

Dear Editors of CoDAS Journal,

The purpose of this letter is to bring some considerations about the challenges and frequent doubts in the elaboration of systematic review (SR) studies, aiming to facilitate the dialogue between research and evidence-based practice (EBP) in Speech-Language Pathology. The SR aims to synthesize data from existing scientific research on a given guiding question with a systematic and explicit method, enabling the presentation of reliable results for decision-making^(1)^. RS is nowadays considered the study with the highest level of scientific evidence.

SR must be differentiated from integrative and scoping reviews, considering the frequent confusion between these methods, including in articles already published, which perpetuates the nomenclature confusion. Integrative reviews are broader studies that allow the inclusion of different designs so that the reader has an overview of a given subject, allowing the understanding of complex concepts, theories, or the health problem targeted by the investigation^(2)^. Formerly considered a type of systematic review^(3)^, the scoping reviews seek to map the evidence available in a given field, analyze knowledge gaps, clarify the main concepts in the literature and the factors related to them, and examine how the research is conducted in a certain field^(4)^. The scoping review has broader and more open questions than systematic reviews and a systematized methodological structure, which differs from the systematic review by the non-mandatory analysis of the risk of bias or methodological quality, and by commonly presenting data synthesis with qualitative character. In this type of review, quantitative syntheses, when performed, refer only to the analysis of the frequency of variables^(3)^.

Another important point is to differentiate the types of SR to be able to correctly plan and execute this research method. The main categories of SR are qualitative evidence, text and opinion, mixed methods, effectiveness, prevalence or incidence, economic evidence, diagnostic test accuracy, etiology and risk, and measurement of psychometric properties^(3)^.

Qualitative SR aims to understand and interpret personal experiences, behaviors, interactions, and social contexts to explain a particular phenomenon of interest^(5)^. Like quantitative research, they require evaluation and criticism, and meta-aggregation can help in this process^(6)^. On the other hand, evidence-based on text and opinion, which can be considered as qualitative research, is drawn from experts' opinions and recommendations, from consensuses, speeches or comments, inferences, or statements by experts on a subject new, or not sufficiently explored in periodicals, journals, monographs, and reports^(7)^. The SR of mixed methods makes it possible to integrate experience (qualitative data) and effectiveness (quantitative data) to identify discrepancies in the available evidence, determine whether quantitative and qualitative data address different aspects of a phenomenon of interest, and explore, contextualize, or explain the findings from the other category of data^(3)^.

Other types of systematic reviews start from a quantitative character and, since the elaboration of their protocol, they need to present a plan concerning the methodology of quantitative evidence synthesis, which allows the generalization of the findings. Effectiveness SR requests to examine the extent to which an intervention, when used properly, achieves its objective^(8)^. The SR of prevalence or incidence describes the distribution of a disease in a given territory, or among subgroups^(4)^. The SR of economic evidence is used to assess intervention effects on health, impacts on resources, associated costs, and added value^(9)^. Another possibility is the SR of diagnostic test accuracy, which allows the comparison of a test of interest or index with an existing one (or reference), to analyze its accuracy^(10)^. The review of etiology and risk seeks to assess the association between different factors to which the individual is exposed and the development of a certain disease or health condition^(11)^. And finally, research on measuring psychometric properties allows self-assessment instruments to be evaluated in terms of their psychometric properties to measure a given construct^(12)^.

An important and specific issue for each SR category is data synthesis, which can be qualitative or quantitative^(13)^. Except in the qualitative systematic reviews mentioned above (qualitative SR, text, and opinion SR, and in some cases of mixed methods SR), meta-analysis (statistical treatment of sample data constituted from the studies that composed the SR) should be planned and described in the protocol. Nevertheless, the feasibility of performing after data extraction is conditioned by several factors such as the number of studies included, the risk of bias, and the heterogeneity between studies. However, it is important to highlight that in the types of SR that it is indicated and there is enough data for its performing, the meta-analysis needs to be carried out, enabling the inference and extrapolation of the results obtained. In cases where it is not possible to perform it, the reason must be described.

**Figure 1:**
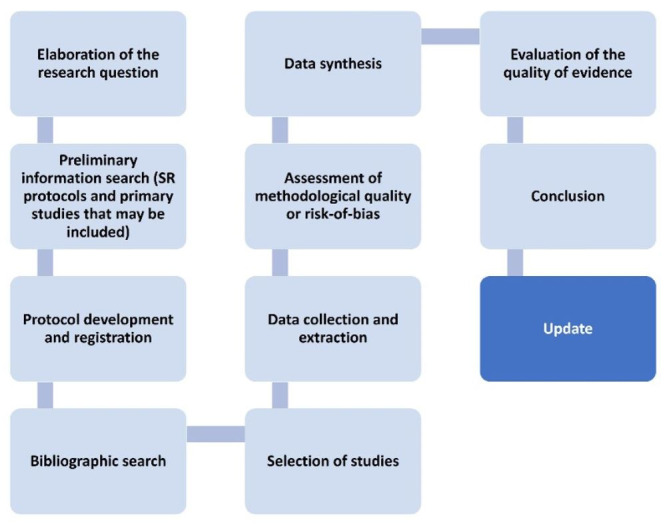
Steps of a Systematic Review

Source: Adapted from Aromataris and Munn^(3)^ and Honório and Santiago^(14)^

Regardless of the type of SR, certain steps necessarily need to be followed, as can be seen in Figure 1. In addition to the ten steps provided for all SR, Cochrane reviews provide for the SR update every five years.

Among the main guidelines and methodological manuals that support the design of a qualified SR can be cited *The Cochrane Reviewer’s Handbook*, *The Australian National Health and Medical Research Council* ^(15)^, and *Joanna Briggs Institute Manual for Evidence Synthesis* ^(3)^. In addition, to help authors improve the reporting of systematic reviews and meta-analysis, the *Preferred Reporting Items for Systematic Reviews and Meta-Analyses* (PRISMA) can be used, which had its version updated in 2020^(16)^.

The research project of an SR is called a protocol, and it must be prepared and registered, *a priori*, before the beginning of the review. The main systematic review registry databases are *The Cochrane Library*; Prospero; *International Prospective Register of Systematic Reviews*; and *Camarades, Collaborative Approach to Meta-Analysis and Review of Animal Data from Experimental Studies*. The register demonstrates transparency in the execution of the research, in addition to avoiding the simultaneous elaboration of studies with the same clinical question^(17)^.

Specifically in Speech-Language Pathology, systematic reviews have revealed that some methodological issues of primary studies also need to be improved to enable the quantitative synthesis of data through meta-analysis. Some of these issues are the need for: I) primary studies that follow design classifications; II) standardization of outcomes and measures; III) a comparison group (whether for intervention, exposure, or outcome); IV) greater methodological detail; and V) complete presentation of quantitative tables.

There are many challenges for the construction of evidence that can support Speech-Language Pathologists practices. However, with the effort, study, and dedication of clinical and academic Speech-Language Pathologists, it is possible and necessary to improve the development and consumption of systematic reviews, which is the opportunity and reason that prompted the preparation of this letter to the Editor.
