# Supplementary material for: Systematic reviews in Speech-Language Pathology
Source: Codas. 2022 Jan 12;34(2):e20210170. doi: 10.1590/2317-1782/20212021170 (PMC9769420; doi:10.1590/2317-1782/20212021170)
Supplement: Translation

# Translation: Revisões sistemáticas em Fonoaudiologia

**Authors:** Vanessa Veis Ribeiro, Carla Patrícia Hernandez Alves Ribeiro César, Raphaela Barroso Guedes-Granzotti, Priscila de Oliveira, Kelly da Silva

**Affiliations:** 1 Departamento de Fonoaudiologia, Universidade Federal de Sergipe – UFS - Lagarto (SE), Brasil.; 2 Departamento de Fonoaudiologia, Universidade Federal de Sergipe – UFS -Aracaju (SE), Brasil.; 3 Departamento de Fonoaudiologia, Universidade Federal da Paraíba – UFPB - João Pessoa (PB), Brasil.

Prezadas editoras da Revista CoDAS,

A proposta desta carta é trazer algumas considerações acerca dos desafios e dúvidas frequentes na elaboração de estudos de Revisão Sistemática (RS), visando facilitar o diálogo entre pesquisas e a prática baseada em evidências (PBE) na Fonoaudiologia. A RS tem como objetivo sintetizar os dados de pesquisas científicas existentes sobre uma determinada pergunta norteadora, com um método sistematizado e explícito, possibilitando a apresentação de resultados confiáveis para a tomada de decisão^(1)^. A RS é considerada hoje o estudo com o mais alto nível de evidência científica.

Para desenvolver este tipo de estudo é necessário diferenciar as RS das revisões integrativas e de escopo, haja visto a frequente confusão destes métodos, inclusive em artigos já publicados, o que acaba perpetuando as confusões de nomenclatura. As revisões integrativas são estudos mais amplos que permitem a inclusão de diferentes tipos de delineamentos para que o leitor tenha um panorama geral sobre um determinado assunto, permitindo compreender conceitos complexos, teorias ou, o problema de saúde alvo da investigação^(2)^. As revisões de escopo, antigamente consideradas como um tipo de revisão sistemática^(3)^, buscam mapear as evidências disponíveis em um determinado campo, analisar lacunas de conhecimento, esclarecer os principais conceitos da literatura e os fatores relacionados a eles e, examinar como a pesquisa é conduzida em uma determinada área^(4)^. A revisão de escopo tem perguntas amplas e mais abertas que as revisões sistemáticas e apresenta uma estrutura metodológica sistematizada, porém, que se diferencia da revisão sistemática pela não obrigatoriedade da análise de risco de viés ou qualidade metodológica e, por comumente apresentar a síntese de dados com caráter qualitativo. Nesse tipo de revisão, sínteses quantitativas, quando realizadas, referem-se apenas à análise de frequência das variáveis^(3)^.

Outro ponto importante é diferenciar os tipos de RS, para que seja possível planejar e executar corretamente este método de pesquisa. Os principais tipos de RS são as de evidências qualitativas, de texto e opinião, de métodos mistos, de efetividade, de prevalência ou incidência, de evidência econômica, de acurácia de teste diagnóstico, de etiologia e risco, e de mensuração de propriedades psicométricas^(3)^.

As RS qualitativas apresentam como objetivo compreender e interpretar experiências pessoais, comportamentos, interações e contextos sociais para explicar um determinado fenômeno de interesse^(5)^. De forma similar às pesquisas quantitativas, requerem avaliação e crítica, sendo que a meta-agregação pode auxiliar nesse processo^(6)^. Já a evidência baseada em texto e opinião (também conhecida como evidência não pesquisada), que pode ser considerada como uma pesquisa qualitativa, é extraída de opiniões e recomendações de especialistas, de consensos, discursos ou comentários, inferências ou afirmações de especialistas sobre um assunto novo ou pouco explorado em periódicos, revistas, monografias e relatórios^(7)^. As RS de métodos mistos possibilitam integrar experiência (dados qualitativos) e eficácia (dados quantitativos) a fim de identificar discrepâncias nas evidências disponíveis, determinar se os dados quantitativos e qualitativos abordam diferentes aspectos de um fenômeno de interesse, e explorar, contextualizar ou explicar as descobertas do outro tipo de dados^(3)^.

Os demais tipos de revisões sistemáticas partem de um caráter quantitativo e, desde a elaboração do seu protocolo, precisam apresentar um planejamento acerca da metodologia de síntese quantitativa de evidência, o que vai permitir a generalização dos achados. A RS de efetividade busca examinar até que ponto uma intervenção, quando utilizada de forma adequada, alcança seu objetivo^(8)^. A RS de prevalência ou incidência descreve a distribuição de uma doença em determinado território, ou entre subgrupos^(4)^. A RS de evidência econômica é utilizada para avaliar efeitos de intervenção na saúde, impactos sobre os recursos, custos associados e valor agregado^(9)^. Outra possibilidade é a RS de acurácia de teste diagnóstico, que permite a comparação de um teste de interesse ou índice com outro já existente (ou de referência), a fim de analisar sua acurácia^(10)^. A revisão de etiologia e risco busca avaliar a associação entre fatores diversos ao que o indivíduo é exposto e o desenvolvimento de uma determinada doença ou condição de saúde^(11)^. E por fim, pesquisas de mensuração de propriedades psicométricas permitem que instrumentos de autoavaliação sejam avaliados quanto às suas propriedades psicométricas para mensurar um determinado construto^(12)^.

Uma questão importante e específica para cada tipo de RS é a síntese de dados, que pode ser qualitativa ou quantitativa^(13)^. Exceto nas revisões sistemáticas de caráter qualitativo citadas acima (RS qualitativa, RS de texto e opinião, e em alguns casos da RS de métodos mistos), a metanálise - tratamento estatístico dos dados da amostra constituída a partir dos estudos que compuseram a RS - deve ser planejada e descrita no protocolo. Porém, a viabilidade de execução após a extração de dados está condicionada a uma série de fatores como a quantidade de estudos incluídos, o risco de viés e a heterogeneidade entre os estudos. Entretanto, é importante destacar que nos tipos de RS que ela é indicada e havendo dados suficientes para a sua realização, a metanálise precisa ser feita, possibilitando a inferência e extrapolação dos resultados obtidos. Nos casos em que não seja possível realizá-la, o motivo deve ser descrito.

Independentemente do tipo de RS, há determinadas etapas que necessariamente precisam ser seguidas, como pode ser observado na Figura 1. Além das dez etapas previstas para todas as RS, nas revisões da Cochrane é prevista a atualização da RS a cada cinco anos.

**Figura 1:** Etapas de uma Revisão Sistemática

Fonte: Adaptado de Aromataris e Munn^(3)^ e Honório e Santiago^(14)^

Dentre as principais diretrizes e manuais metodológicos que auxiliam no delineamento de uma RS de qualidade podem ser citados *The Cochrane Reviewer’s Handbook*, *The Australian National Health and Medical Research Council* ^(15)^ e o *Joanna Briggs Institute Manual for Evidence Synthesis* ^(3)^. Além disso, para ajudar os autores a melhorar o relato das revisões sistemáticas e metanálise pode ser utilizado o *Preferred Reporting Items for Systematic Reviews and Meta-Analyses* (PRISMA), que teve sua versão atualizada em 2020^(16)^.

O projeto de pesquisa de uma RS é denominado protocolo, devendo ser elaborado e registrado, a priori, antes do início da revisão. As principais bases de registros de revisões sistemáticas são: *The Cochrane Library*; Prospero; *International Prospective Register of Systematic Reviews*; e *Camarades, Collaborative Approach to Meta-Analysis and Review of Animal Data from Experimental Studies*. O registro demonstra a transparência na execução da pesquisa, além de evitar a elaboração concomitante de estudos com a mesma pergunta clínica^(17)^.

Especificamente na Fonoaudiologia, as revisões sistemáticas têm revelado que algumas questões metodológicas dos estudos primários também precisam ser aprimoradas para possibilitar a síntese quantitativa de dados por meio de metanálise. Algumas dessas questões são a necessidade de: I) estudos primários que sigam classificações de delineamento; II) padronização de desfechos e medidas; III) necessidade de grupo de comparação (seja ele de intervenção, exposição ou desfecho); IV) maior detalhamento metodológico; e, V) apresentação completa de tabelas quantitativas.

Muitos são os desafios para a construção de evidências que possam subsidiar as práticas fonoaudiológicas. Porém, com esforço, estudo e dedicação dos fonoaudiólogos do meio clínico e acadêmico, é possível e necessário melhorar o desenvolvimento e o consumo de revisões sistemáticas, sendo esse o ensejo e o motivo que impulsionou a elaboração desta carta ao Editor.
